# Taxonomic update on the genus *Unihamus* Luo & Wang, 2025 (Blattidae, Blattinae), with three new species described

**DOI:** 10.3897/zookeys.1277.163465

**Published:** 2026-04-16

**Authors:** Xin-Xing Luo, Meng-Yu Qiu, Ting-Ting Cai, Zong-Qing Wang, Yan-Li Che

**Affiliations:** 1 College of Plant Protection, Southwest University, Beibei, Chongqing 400715, China College of Plant Protection, Southwest University Chongqing China; 2 Key Laboratory of Agricultural Biosafety and Green Production of Upper Yangtze River (Ministry of Education), Southwest University, Chongqing 400715, China Key Laboratory of Agricultural Biosafety and Green Production of Upper Yangtze River (Ministry of Education), Southwest University Chongqing China

**Keywords:** DNA barcoding, new species, *Periplaneta* sensu lato, sclerite L3, *
Unihamus
*

## Abstract

*Unihamus* Luo & Wang, 2025 was recently split from *Periplaneta* sensu lato, yet its diversity and morphology remain poorly documented. Here we redescribe the type, *Unihamus elegans* (Hanitsch, 1927) and describe three new species, *U. flavus* Luo & Che, **sp. nov**., *U. concavus* Luo & Che, **sp. nov**., and *U. longispinus* Luo & Che, **sp. nov**. Males and females are associated by using DNA barcoding. We present high-resolution photographs and detailed diagnoses for each species. Finally, the future research directions of the distinctive sclerite L3 were prospected.

## Introduction

*Unihamus elegans* (Hanitsch, 1927) was originally described from Lam Dong Province, Vietnam, as a member of the genus *Periplaneta* Burmeister, 1838. Later, this species was reported from Baoshan, China (Bey-Bienko 1957). However, combining morphological and molecular evidence, Luo et al. (2025) transferred Chinese species of *Periplaneta* to several newly erected genera, including *Unihamus* Luo & Wang, 2025. This genus currently comprised three taxa: *U. elegans* (Hanitsch, 1927), and two undescribed congeners (*Unihamus* sp. 1 and *Unihamus* sp. 2). Although *U. elegans* was long treated as a member of *Periplaneta*, Luo et al. (2025) in their phylogenetic study recovered *U. elegans* and two unnamed species (sp. 1 and sp. 2) as the sister group of *Eroblatta* Shelford, 1910, far removed from *Periplaneta* sensu stricto. In external morphology, *Unihamus* is scarcely distinguishable from *Periplaneta* s.s. and the other blattine genera erected by Luo et al. (2025). However, male genitalia provide reliable diagnostic characters: in particular, sclerite L3 in *Unihamus* is markedly different from that of all other blattine genera, and the remaining sclerites also show distinctive configurations. Luo et al. (2025) made a simple diagnosis of *Unihamus*, distinguishing from *Periplaneta* s.s. and other genera, but they did not provide a detailed species-level description for *Unihamus*.

We redescribe *U. elegans* in detail here and describe three new species from China, including *Unihamus* sp. 1, *Unihamus* sp. 2 mentioned in Luo et al. (2025). We pair males and females using COI barcoding and provide detailed descriptions, including the genital characters, of these species.

## Materials and methods

### Specimen sources and treatment

Unless otherwise specified, specimens were collected in Hainan, Guangxi and Yunnan provinces, China, and deposited in College of Plant Protection, Southwest University (**SWU**), Chongqing, China. Procedures for specimen preservation, morphological dissection, photography, DNA extraction, amplification, and sequencing followed the protocols of Luo et al. (2023).

### Morphological terminology

Terminology for external morphology and wing venation follows Roth (2003) and Li et al. (2018), whereas genital terminology for males and females follows Klass (1997) and McKittrick (1964), respectively.

The abbreviations employed in this study are as follows. Veins: cubitus (**Cu**), cubitus anterior (**CuA**), cubitus posterior (**CuP**), media (**M**), postcubitus (**Pcu**), radius (**R**), radius anterior (**RA**), radius posterior, (**RP**), subcostal posterior (**ScP**), vannal veins (**V**). Male genitalia: sclerites of left phallomere (**L1**, **L2**, **L3**, **L4C**, **L4D**, **L4E**, **L4G**), sclerites of right phallomere (**R1G**, **R1H**, **R1F**, **R2**, **R3**). Female genitalia: tergum X (**TX**), first valve (**v.I.**), first valvifer (**vlf.I**), second valve (**v.II**), posterior lobes of valvifer II (**p.l.**), laterosternite IX (**ltst.IX**), anterior arch (**a.a.**), spermathecal (**sp**.), spermathecal plate (**sp.pl.**), spermathecal opening (**sp.o.**), basivalvulae (**bsv.**), laterosternal shelf (**ltst.sh.**).

### Sequence processing and molecular analyses

We analysed 40 COI sequences: 38 ingroup sequences (four species of *Unihamus* and 22 species from other blattine genera) and two outgroup sequences, *Cryptocercus meridianus* Grandcolas & Legendre, 2005 and *Cryptocercus punctulatus* Scudder, 1862 (Suppl. material 1). Sequences were aligned by using the MUSCLE algorithm implemented in MEGA v. 11 (Kumar et al. 2016) and adjusted by translating into amino acid. Pairwise genetic distances were calculated using the Kimura 2-parameter (K2P) distance model in MEGA v. 11 (Kimura 1980). The optimal partitioning scheme and best-fit substitution models for each codon position of COI (COI_pos 1: TRN+G; COI_pos 2: K81UF+I+G; COI_pos 3: TVM+G) were selected with PartitionFinder v. 2.1.1 (Lanfear et al. 2017) using the corrected Akaike Information Criterion (AICc). A maximum-likelihood (ML) analysis was performed in IQ-TREE v. 2.2.0 (Nguyen et al. 2015) on the partitioned dataset; node supports were assessed by 10,000 ultrafast bootstrap (UFBoot) replicates, and the “-bnni” option was applied to reduce model violations.

## Results

### Genus Unihamus

Taxon classificationAnimaliaBlattodeaBlattidae

Luo & Wang, 2025

9254BDCF-31E6-537A-A6E8-7EF4AD16F9A1

Unihamus Luo & Wang in Luo et al. 2025. Type species: Periplaneta elegans Hanitsch, 1927, by original designation.

#### Generic diagnosis.

Body medium-sized. Sexual dimorphism weak: female broader than male; tegmina and wings of female shorter than male, but all surpassing tip of abdomen. **Male**. Antennae longer than body. Pronotum subelliptical, widest point posterior to midpoint. Posterior margin of metanotum mesally produced. Tegmina ScP slightly thickened. Front femur of type A_2_. Pulvilli present; claws symmetrical and unspecialized; arolium medium-sized. Hind metatarsus longer than or equal to remaining tarsomeres combined; metatarsi with two rows of spinules ventrally; each side of pulvillus with one spinule. First abdominal tergite with visible, setose gland. Middle part of hind margin of supra-anal plate and subgenital plate concave inwards. L3 unciform and not bifurcated. L4C with process near basal right margin. Distal part of R1G with two spines. R2 large and complex. Anterior margin of R3 triangular. **Female**. Outer margin of posterior lobes of valvifer II merged with laterosternite IX.

The simple antenna of *Unihamus* differ from that of *Thyrsocera* Burmeister, 1838, which has the antenna plumose or with plumose portions in males and moniliform in females.

This macropterous genus can be readily distinguished from the apterous and micropterous blattid genera (i.e. *Afrostylopyga* Anisyutkin, 2014, *Apterisca* Princis, 1963, *Brinckella* Princis, 1963, *Henicotyle* Rehn & Hebard, 1927, *Macrostylopyga* Anisyutkin et al., 2013, *Miostylopyga* Princis, 1966, and *Neostylopyga* Shelford, 1911). The weak sexual dimorphism (macroptery of males and females) in *Unihamus* differs from distinct sexual dimorphism in other genera:

- Macropterous male and apterous female; i.e. *Deropeltis* Burmeister, 1838, *Archiblatta* Snellen van Vollenhoven, 1862, and *Catara* Walker, 1868);
- Macropterous male and micropterous female; i.e. *Blatta* Linnaeus, 1758, *Planiblatta* Luo & Wang, 2023, *Pseudoderopeltis* Krauss, 1890);
- Macropterous male and brachypterous female; i.e. *Arcicaulis* Luo & Wang, 2025, *Bundoksia* Lucañas, 2021, *Cartoblatta* Shelford, 1910, *Tenumembrana* Luo & Wang, 2025, some *Periplaneta* Burmeister, 1838, *Protagonista* Shelford, 1908, *Scabinopsis* Bey-Bienko, 1969, and *Vittiblatta* Luo & Wang, 2023).

The hind metatarsus of *Unihamus* is longer than or equal to the remaining tarsal segments combined, which differs from *Atemeleta* Brunner von Wattenwyl, 1893, *Eumethana* Princis, 1951, and *Scabinopsis*.

The first abdominal tergite has a visible, setose gland in *Unihamus*. Setose glands are absent in *Blatta*, *Cartoblatta*, *Homalosilpha* Stål, 1874, *Periplaneta* s.s., and *Valaraukar* Lucañas, 2025.

Species of *Unihamus* in which L3 is not bifurcated can be distinguished from other genera with a bifurcated L3. These genera are *Archiblatta*, *Arcicaulis*, *Blatta*, *Bundoksia*, *Catara*, *Crescispina* Luo & Wang, 2025, *Deropeltis*, *Dorylaea* Stål, 1877, *Eroblatta* Shelford, 1910, *Hobbitoblatta* Lucañas, 2023, *Homalosilpha*, *Nazgultaure* Lucañas, 2023, *Tenumembrana*, *Valaraukar*, *Validiblatta* Luo & Wang, 2025, and *Vittiblatta*.

##### Key to males of known species of *Unihamus*

**Table:** 

1	Male supra-anal plate W-shaped; distal part of L2 with two spines (Fig. 5F, H)	***U. concavus* Luo & Che, sp. nov**.
–	Male supra-anal plate not W-shaped, and the distal part of L2 with a spine	**2**
2	Distal part of L2 longer than L3 (Figs 6H, 7G)	***U. longispinus* Luo & Che, sp. nov**.
–	Distal part of L2 not longer than L3	**3**
3	Hind margin of male supra-anal plate with small, sparse spines; central part of ventral surface with spiny, transverse projections (Fig. 3F)	***U. elegans***
–	Hind margin of male supra-anal plate without spine; central part of ventral surface without projections (Fig. 4F)	***U. flavus* Luo & Che, sp. nov**.

### Unihamus elegans

Taxon classificationAnimaliaBlattodeaBlattidae

(Hanitsch, 1927)

9AD71B2C-A63E-5182-B8C9-5A91EDA52B09

**Figure 1.**
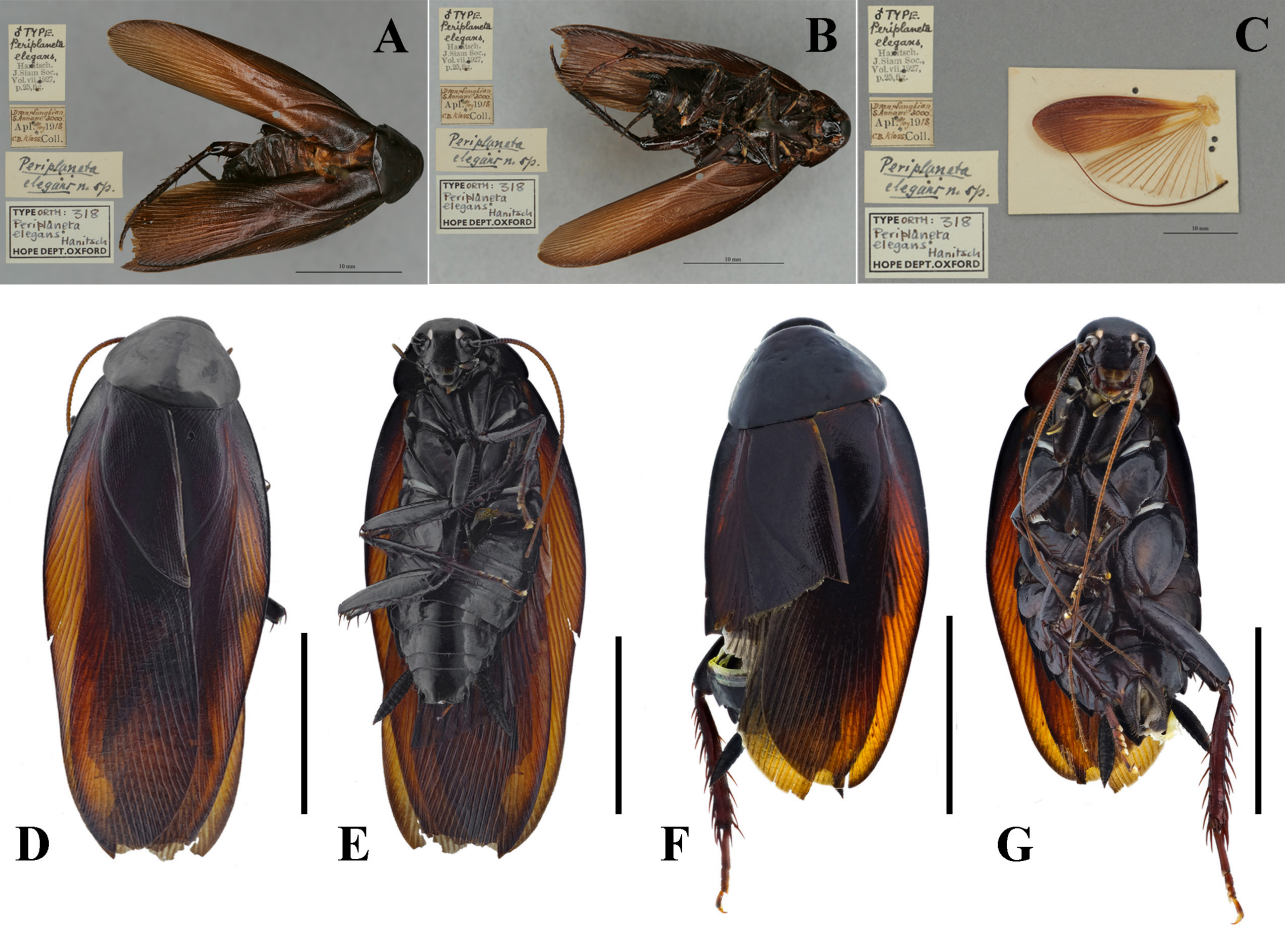
*Unihamus elegans* (Hanitsch, 1927). **A–C**. Holotype (male); **A**. Dorsal view; **B**. Ventral view; **C**. Hind wing; **D**. Male, dorsal view; **E**. Male, ventral view; **F**. Female, dorsal view; **G**. Female, ventral view. Scale bars: 10 mm (**D–G**). (**A–C**. Photographed by Robert Douglas, Oxford University Museum of Natural History; **D–G**. Photographed by the authors, based on specimens deposited in the College of Plant Protection, Southwest University).

Figs 1, 3, 7A, B, 8A, E, I, 9A

Periplaneta elegans Hanitsch, 1927: 25 (holotype: ♂, Vietnam; OUMNH; England); Bey-Bienko 1957: 896; Princis 1966: 445.
Unihamus elegans : Luo et al. 2025: 844.

#### Material examined.

**Holotype** • ♂ (OUM, TYPE ENT-ORTH 318) Vietnam: “♂ TYPE. *Periplaneta elegans*, Hanitsch. J. Siam Soc., Vol. vii, 1927, p. 25, fig.”; “Dran, Langbian, S. Annam, 3,000'. Apl. May 1918. CB. Kloss. Coll.”, “*Periplaneta elegans* n. sp.”; “TYPE ORTH: 318 *Periplaneta elegans* Hanitsch / HOPE DEPT. OXFORD”.

**Figure 2.**
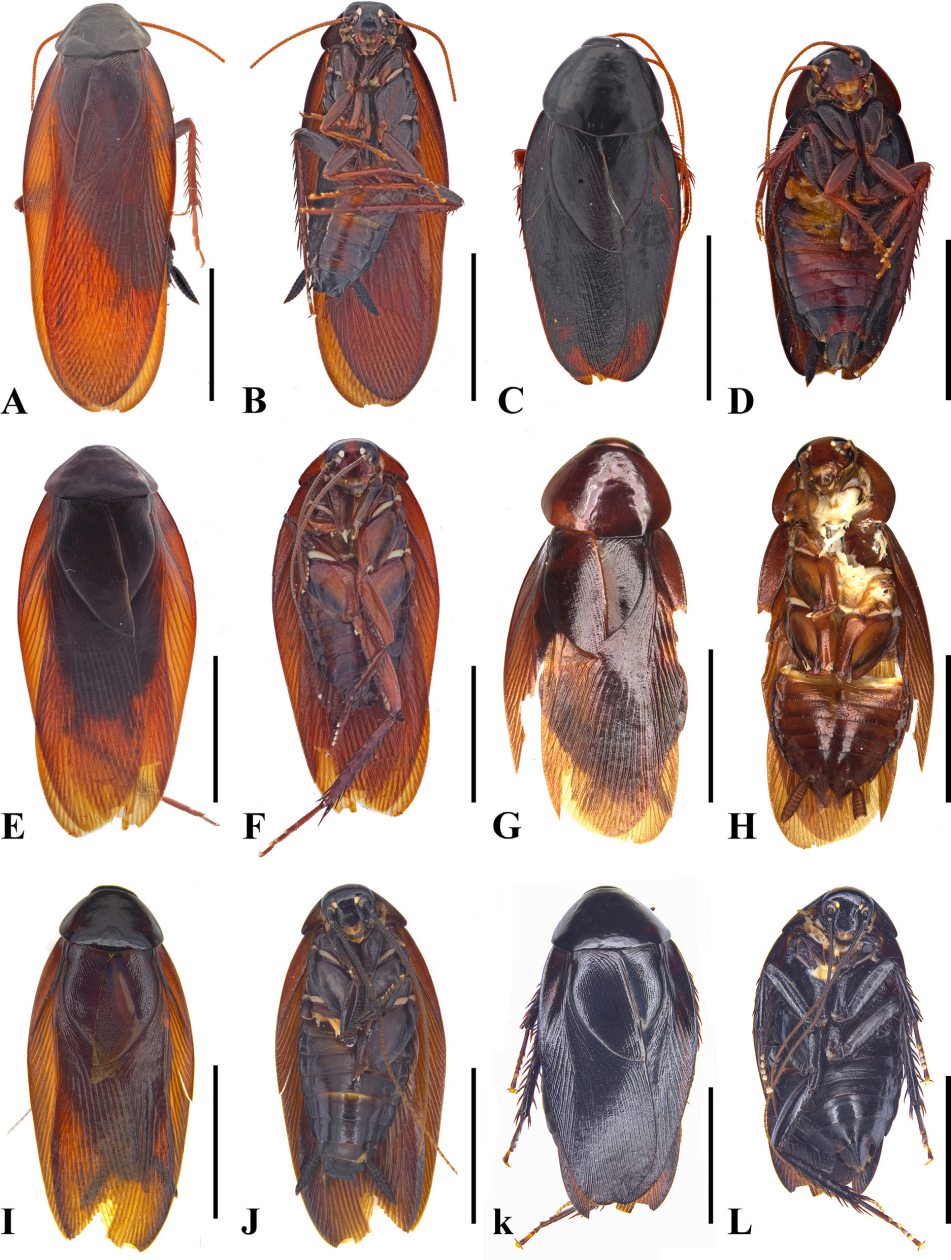
Habitus of *Unihamus* spp. **A–D**. *U. flavus* Luo & Che, sp. nov.; **A**. Male holotype, dorsal view; **B**. Male holotype, ventral view; **C**. Female paratype, dorsal view; **D**. Female paratype, ventral view; **E–H**. *U. concavus* Luo & Che, sp. nov.: **E**. Male holotype, dorsal view; **F**. Male holotype, ventral view; **G**. Female paratype, dorsal view; **H**. Female paratype, ventral view; **I–L**. *U. longispinus* Luo & Che, sp. nov.: **I**. Male holotype, dorsal view; **J**. Male holotype, ventral view; **K**. Female paratype, dorsal view; **L**. Female paratype, ventral view. Scale bars: 10 mm.

China • 2♂♂, 1♀; Yunnan, Butterfly Valley, Maandi village, Jinping County; 14–16.V.2015; Jianyue Qiu leg; SWU-B-BL-080301 to 080303 • 2♀♀; Yunnan, Daweishan Mountain, Pingbian County; 16–17.V.2015; Chenglong Ren & Jianyue Qiu leg.; SWU-B-BL-080304 to 080305.

#### Diagnosis.

Vertex exposed. Pronotum subelliptical, widest point posterior to midpoint. Posterior margin of metanotum mesally produced. Tegmina and wings well developed. Posterior branch of R not reaching end of tegmina. Hind metatarsus longer than remaining tarsomeres combined. First abdominal tergite with visible gland. Surface of supra-anal plate with small, sparse spines; median of hind margin incised; middle part of ventral surface with transverse projection, covered with small spines. Hind margin of subgenital plate slightly concave. L1 weakly sclerotized. L2 with long ventral spine pointing inward. L3 not bifurcate. L4C lanceolate, with blade-like process. Left margin of R1H extending into two spines; ventral surface with a spine. Right ventral surface of R1G with a finger-like spine and another long spine.

**Figure 3.**
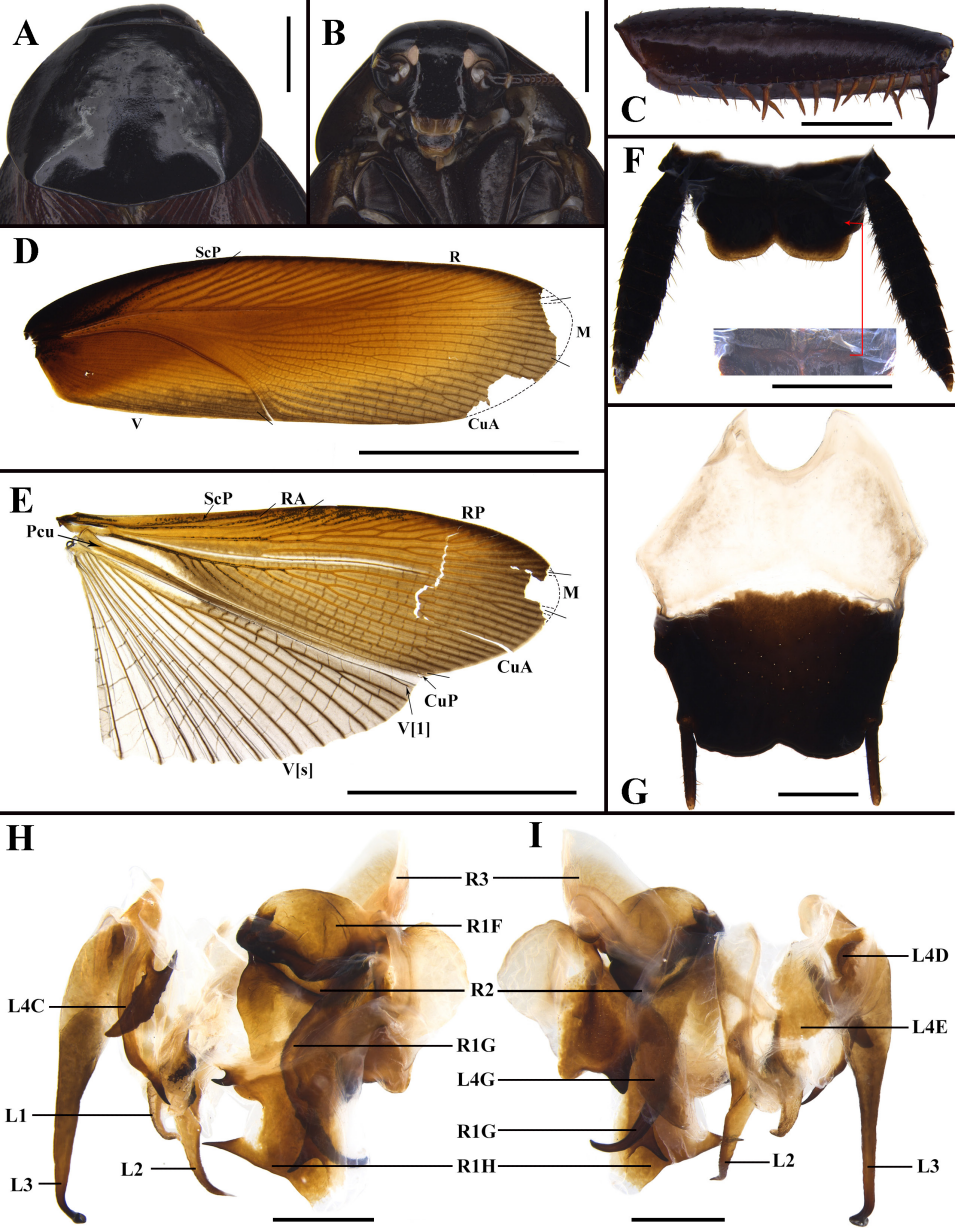
*Unihamus elegans* (Hanitsch, 1927) male. **A**. Pronotum, dorsal view; **B**. Head, ventral view; **C**. Front femur, ventral view; **D**. Tegmen, dorsal view; **E**. Hind wing, dorsal view; **F**. Supra-anal plate and transverse projection, ventral view; **G**. Subgenital plate, dorsal view; **H, I**. Male genitalia (dorsal and ventral view). Scale bars: 2 mm (**A, B, F**); 1 mm (**C, G–I**); 10 mm (**D, E**).

**Figure 4.**
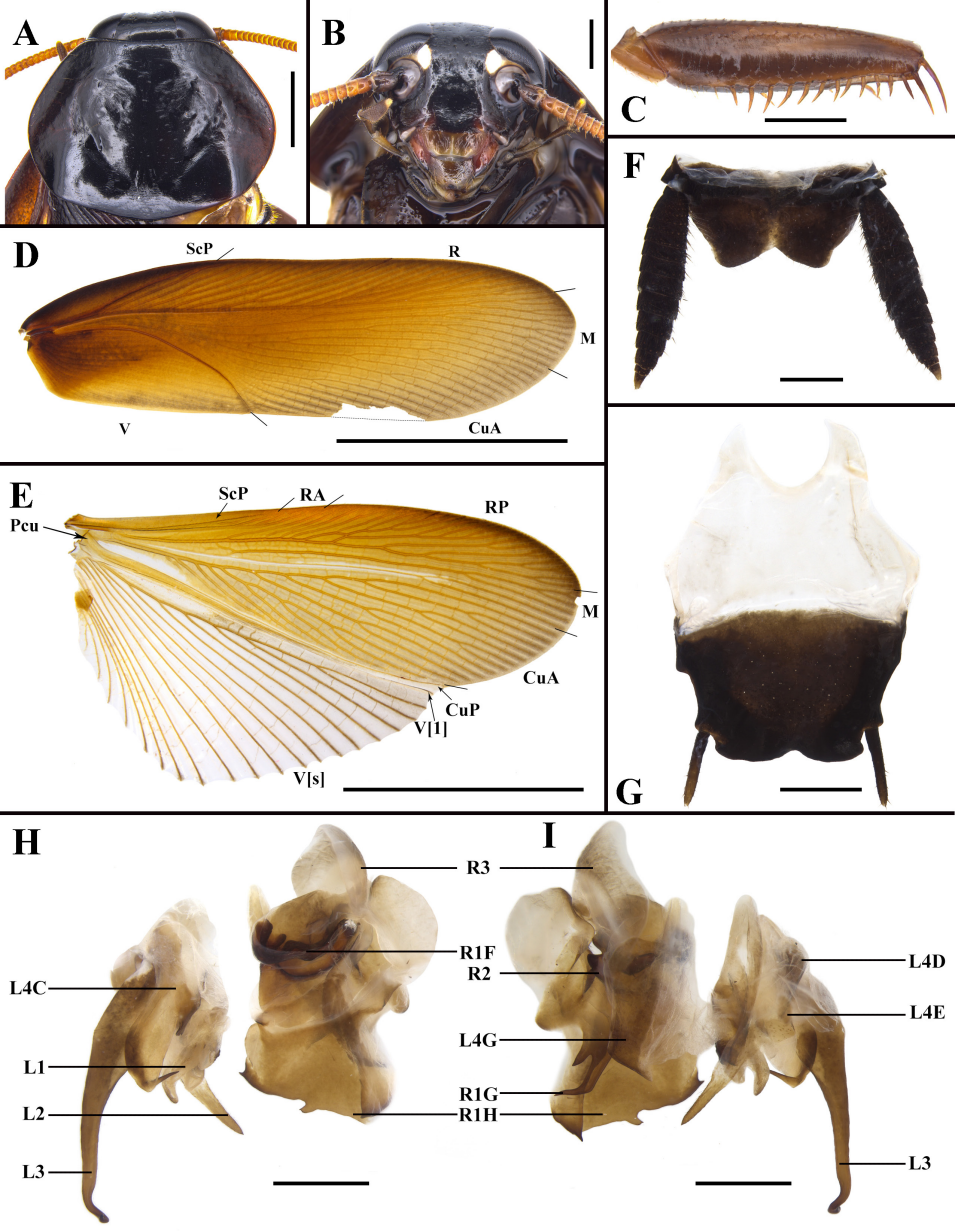
*Unihamus flavus* Luo & Che, sp. nov. (male holotype). **A**. Pronotum, dorsal view; **B**. Head, ventral view; **C**. Front femur, ventral view; **D**. Tegmen, dorsal view; **E**. Hind wing, dorsal view; **F**. Supra-anal plate, ventral view; **G**. Subgenital plate, dorsal view; **H, I**. Male genitalia (dorsal and ventral view). Scale bars: 2.0 mm (**A**); 1.0 mm (**B, C, F–I**); 10.0 mm (**D, E**).

This species can be easily distinguished from congeners by the following characters: 1) ventral surface of supra-anal plate with transverse, striate projection covered with small spines (Fig. 3F); 2) acute blade-like process present near basal right margin of L4C (Figs 3H, 7A); 3) L4C lanceolate and not curved (differs from *U. flavus* and *U. concavus*) (Figs 4H, 7A); 4) R1H broad (differs from *U. longispinus*) (Figs 3H, 7B).

#### Redescription.

***Colouration***. Body black, except: ocelli white, antennae brown, tegmina reddish brown, tarsi blackish brown.

**Male**. Total length: 27.2–27.5 mm; body length: 18.5 mm; pronotum length × width: 4.8–5.2 × 5.7–6.1 mm; tegmina length × width: 22.0–22.3 × 6.6–6.7 mm. ***Head and thorax***. Vertex exposed; interocular space almost equal to interocellar space, shorter than interantennal space (Fig. 3B). Pronotum subelliptical, with anterior margin nearly straight and hind margin convex, surface smooth, widest point posterior to midpoint (Fig. 3A). Posterior margin of metanotum mesally produced; posterior-lateral angles with symmetrical projections (Fig. 8A). Tegmina and wings well developed, surpassing tip of abdomen (Figs 1D, 1E, 3D, 3E). Tegmina ScP slightly thickened; posterior branch of R not reaching end of tegmina (Fig. 3D). Front femur of type A_2_ (Fig. 3C). Hind metatarsus longer than remaining tarsomeres combined; metatarsi with two rows of spinules ventrally and one spinule on each side of pulvillus. Pulvilli present; claws symmetrical and unspecialized; arolium medium-sized. ***Abdomen***. First abdominal tergite gland with downward-pointing setae (Fig. 8A). Lateral margin of supra-anal plate contracted inward near distal part and less sclerotized at margin; surface with small, sparse spines; middle of hind margin incised and divided into two subobtusely triangular lobules; middle part of ventral surface with transverse projection, covered with small spines (Fig. 3F). Paraprocts long and strip-like. Cerci strong and symmetrical. Subgenital plate overall nearly square; hind margin of interstylar slightly concave (Fig. 3G). ***Genitalia*** (Figs 3H, 3I, 7A, 7B, 8E). L1 weakly sclerotized, bearing pubescence (Fig. 7A). L2 irregular, with long, ventral, inward-pointing spine (Fig. 7A). L3 unciform and well sclerotized, not bifurcate (Figs 7A, 8E). L4C lanceolate, with distally acute, blade-like process near inner basal margin (Fig. 7A). R1H broad and irregular; left margin extending into two spines; ventral surface with a spine (Fig. 7B). R1G well sclerotized; right side of ventral surface with a finger-like spine and another long spine (Figs 3I, 7B).

**Figure 5.**
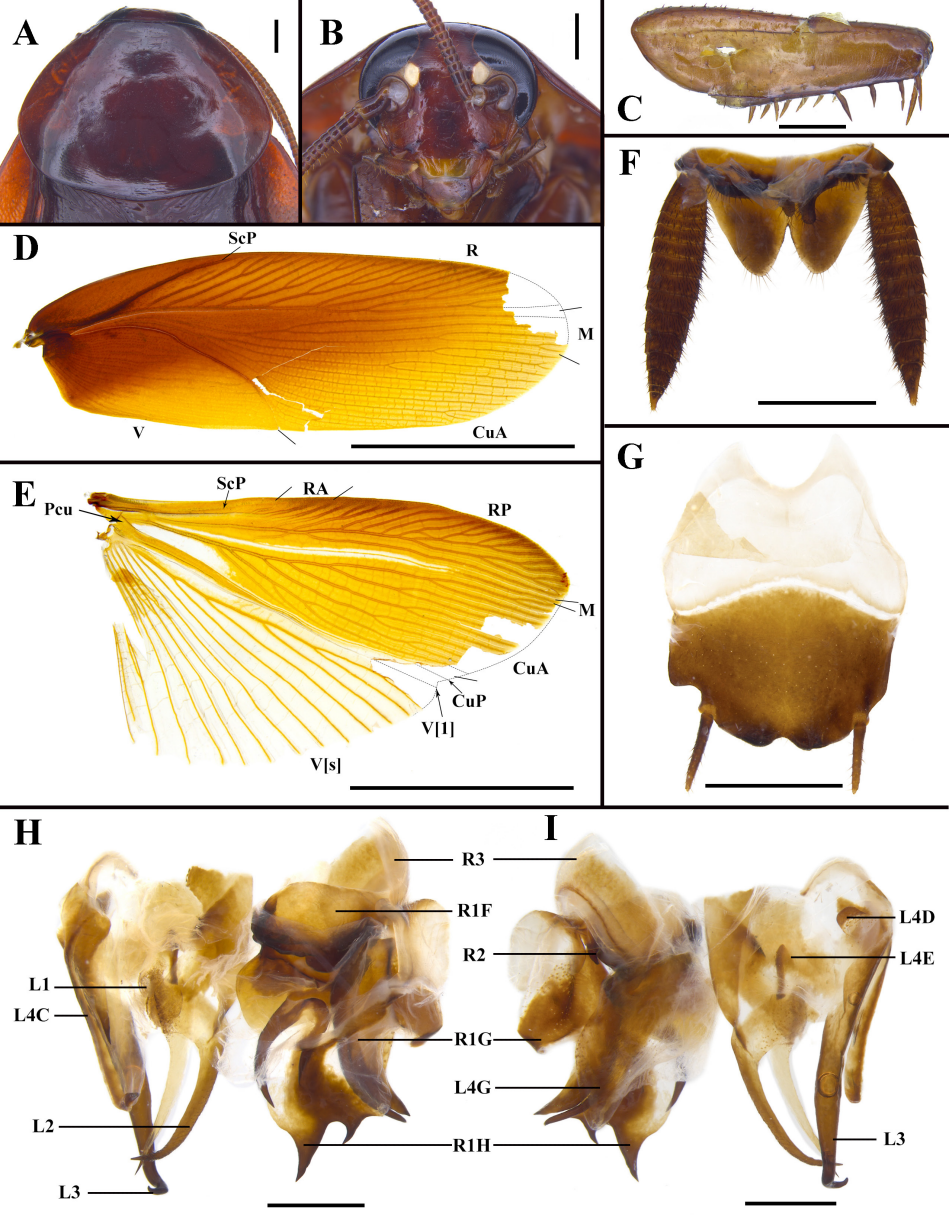
*Unihamus concavus* Luo & Che, sp. nov. (male holotype). **A**. Pronotum, dorsal view; **B**. Head, ventral view; **C**. Front femur, ventral view; **D**. Tegmen, dorsal view; **E**. Hind wing, dorsal view; **F**. Supra-anal plate, ventral view; **G**. Subgenital plate, dorsal view; **H, I**. Male genitalia (dorsal and ventral view). Scale bars: 10.0 mm (**D, E**); 2.0 mm (**F, G**); 1.0 mm (**A–C, H, I**).

**Figure 6.**
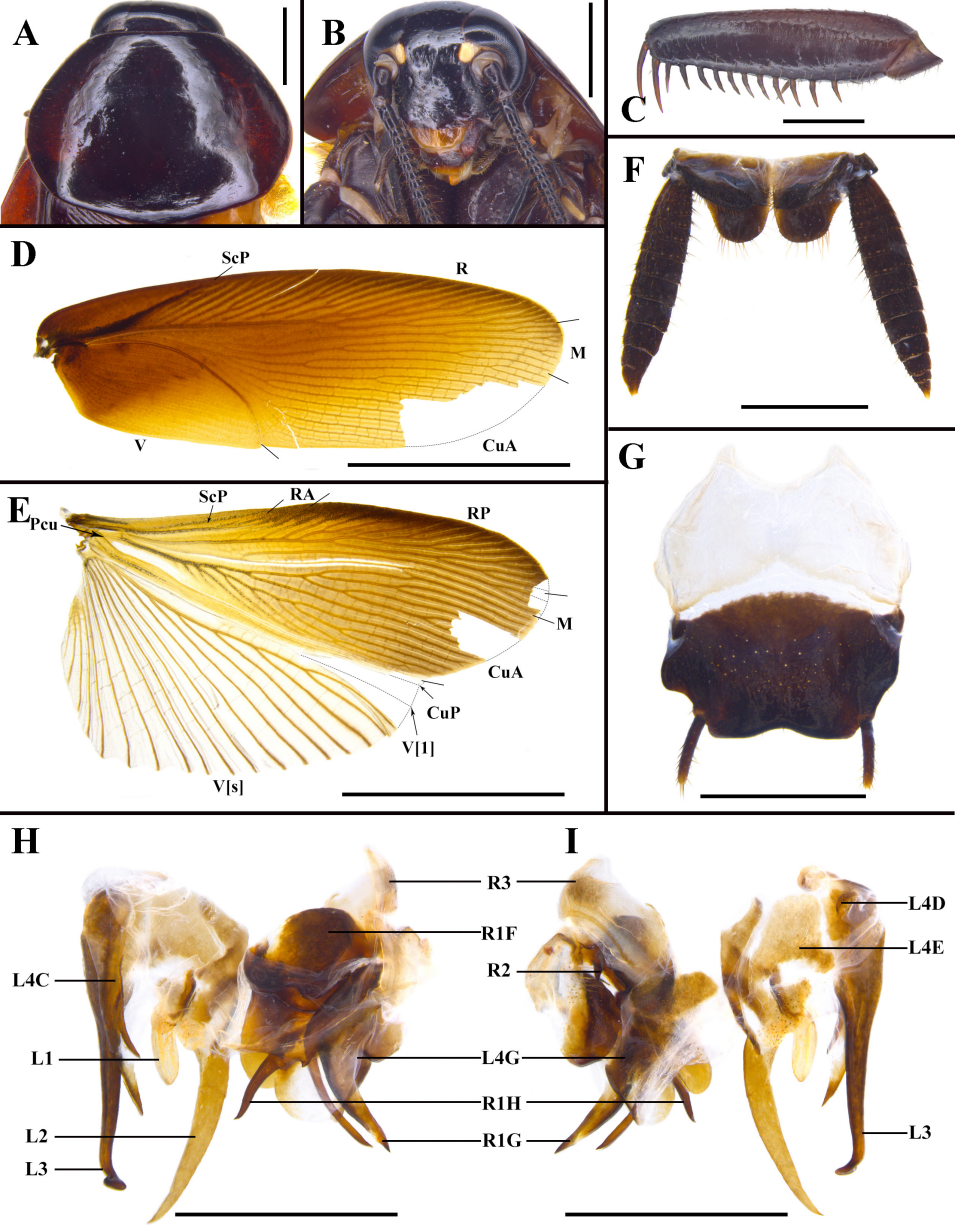
*Unihamus longispinus* Luo & Che, sp. nov. (male holotype). **A**. Pronotum, dorsal view; **B**. Head, ventral view; **C**. Front femur, ventral view; **D**. Tegmen, dorsal view; **E**. Hind wing, dorsal view; **F**. Supra-anal plate, ventral view; **G**. Subgenital plate, dorsal view; **H, I**. Male genitalia (dorsal and ventral view). Scale bars: 2.0 mm (**A, B, F–I**); 1.0 mm (**C**); 10.0 mm (**D, E**).

**Figure 7.**
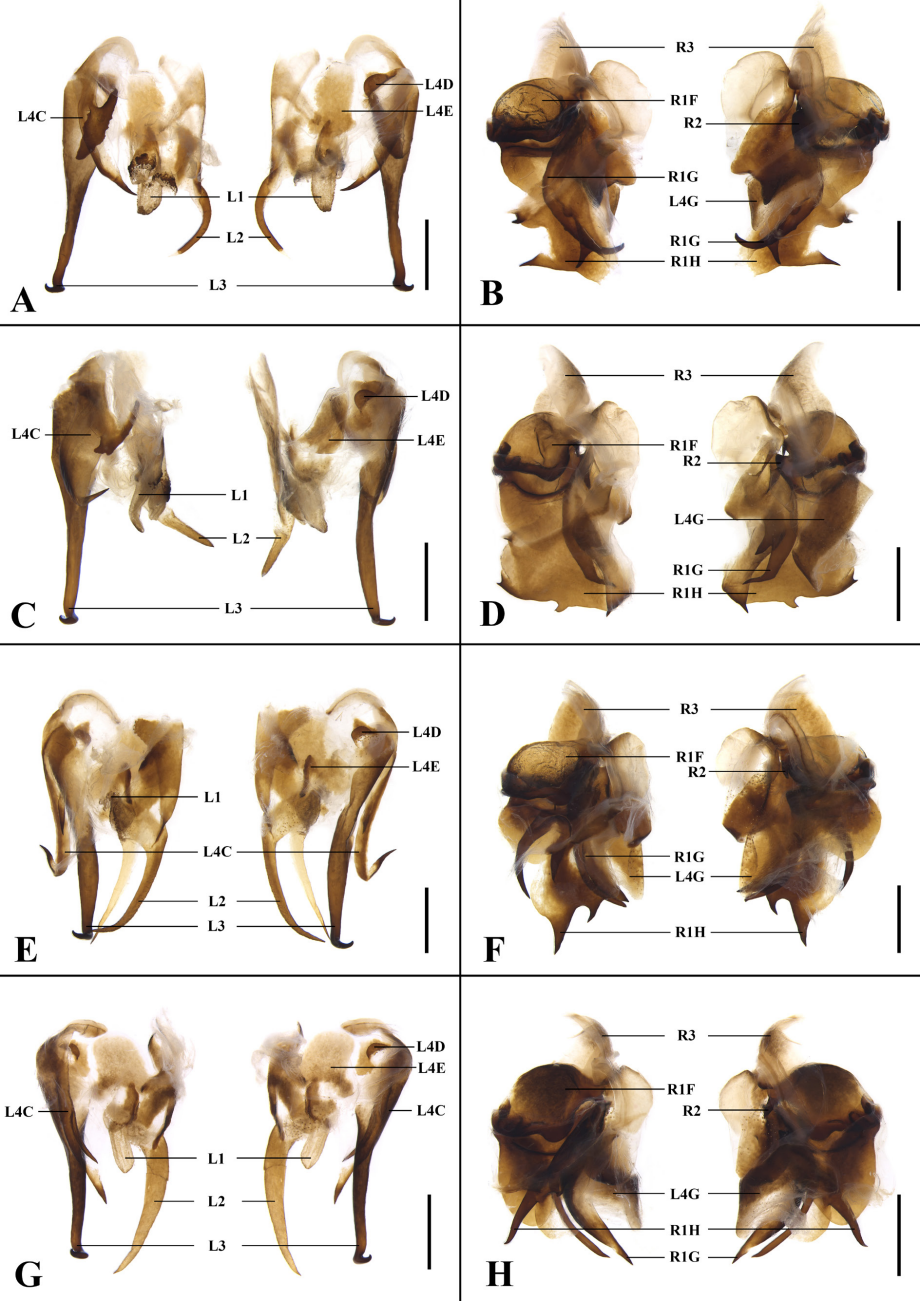
Details of male genitalia. In order from left to right: left phallomere, dorsal and ventral views; right phallomere, dorsal and ventral views. **A, B**. *Unihamus elegans* (Hanitsch, 1927); **C, D**. *Unihamus flavus* Luo & Che, sp. nov. (holotype); **E, F**. *Unihamus concavus* Luo & Che, sp. nov. (holotype); **G, H**. *Unihamus longispinus* Luo & Che, sp. nov. (holotype). Scale bars: 1.0 mm.

**Figure 8.**
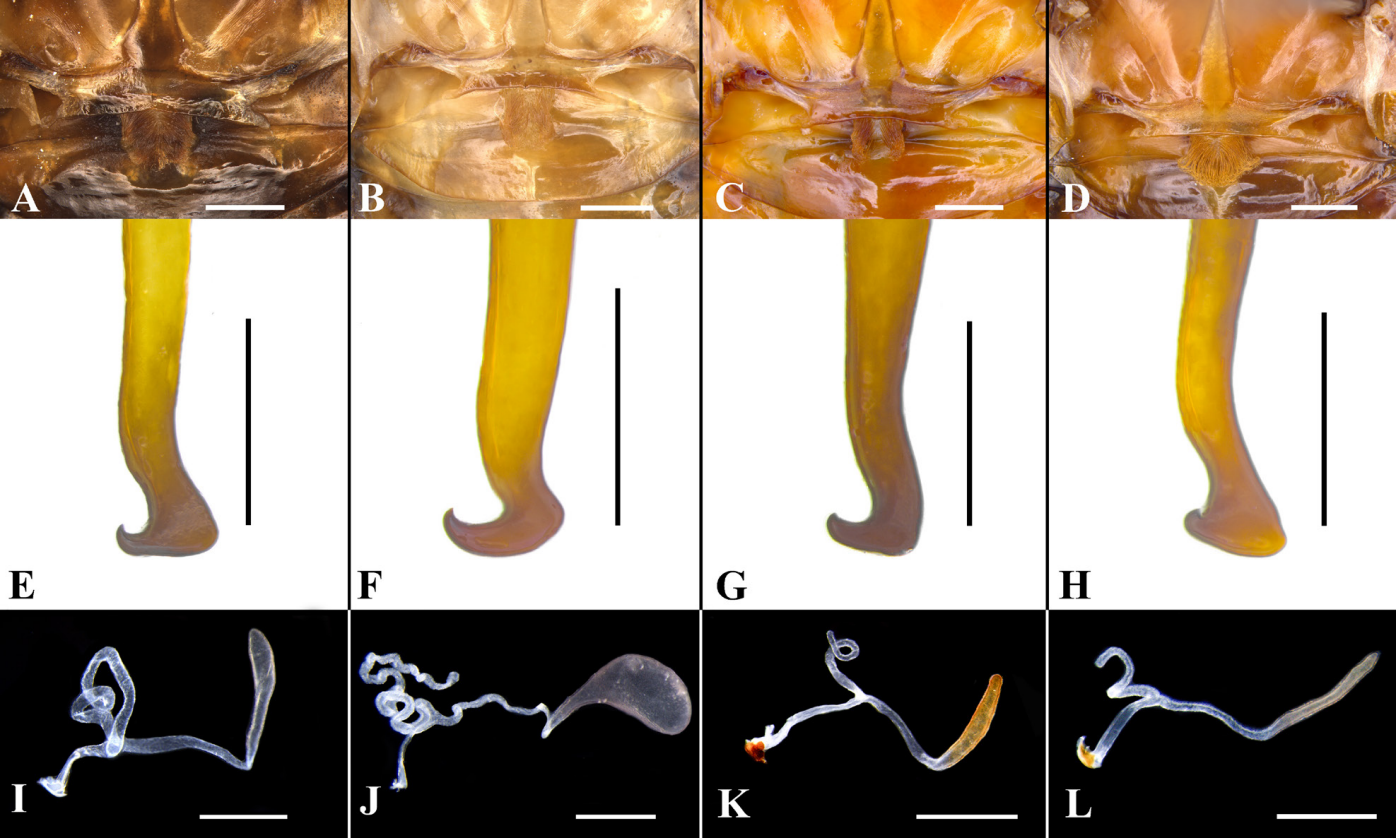
**A, E**. *Unihamus elegans* (Hanitsch, 1927) (male); **B, F**. *Unihamus flavus* Luo & Che, sp. nov. (male holotype); **C, G**. *Unihamus concavus* Luo & Che, sp. nov. (male holotype); **D, H**. *Unihamus longispinus* Luo & Che, sp. nov. (male holotype); **A–D**. Hind margin of metanotum and tergal gland; **E–H**. Hla (sclerite L3); **I**. *Unihamus elegans* (Hanitsch, 1927); **J**. *Unihamus flavus* Luo & Che, sp. nov. (paratype); **K**. *Unihamus concavus* Luo & Che, sp. nov. (paratype); **L**. *Unihamus longispinus* Luo & Che, sp. nov. (paratype); **I–L**. Female spermatheca. Scale bars: 1.0 mm (**A–D**); 0.5 mm (**E–L**).

**Figure 9.**
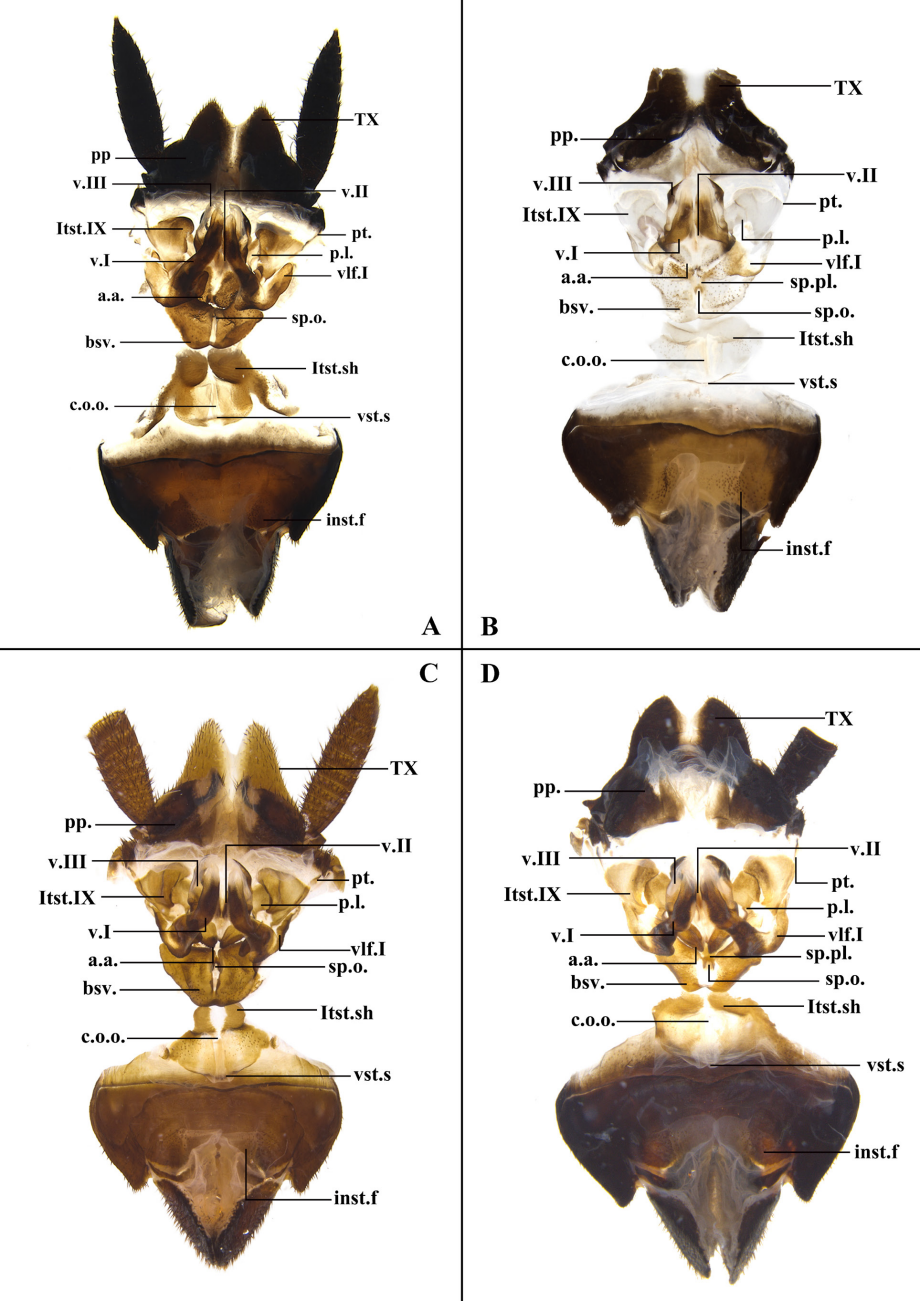
Female genitalia. **A**. *Unihamus elegans* (Hanitsch, 1927); **B**. *Unihamus flavus* Luo & Che, sp. nov. (paratype); **C**. *Unihamus concavus* Luo & Che, sp. nov. (paratype); **D**. *Unihamus longispinus* Luo & Che, sp. nov. (paratype). Scale bars: 2.0 mm.

**Female** (Figs 1F, 1G, 8I, 9A). Body broader than male. Total length: 23.1–28.1 mm; body length: 20.5–25.0 mm; pronotum length × width: 6.0–6.4 × 7.4–8.0 mm; tegmina length × width: 17.7–23.2 × 6.3–8.1 mm. ***Head and thorax***. Same as in male. ***Abdomen***. Hind margin of tergum X with median invagination and a membrane line inside (Fig. 9A). ***Genitalia*** (Fig. 9A). First valve sclerotized, with small, dense punctures, and basal part with microtrichia; left and right valve asymmetrical; right margin of left valve with a rhombic projection. First valvifer broad and slightly curved. Second valve strip-like. Third valve large and wrapping around second valve. Posterior lobes of valvifer II less sclerotized and outer margin connected to irregular laterosternite IX. Paratergites long, strip-like, and slender. Anterior arch nearly rectangular and outer and hind margin with small, dense spines. Spermathecal plate somewhat fan-shaped, connected to basivalvula by membrane. Spermathecal opening located at middle of basivalvula. Spermatheca branched into a leading duct and branching duct, with duct short and end capsule rod-shaped (Fig. 8I). Basivalvulae (bsv.) nearly rectangular, asymmetrical, with microtrichia. Laterosternal shelf (ltst.sh.) symmetrical, with sparse punctures.

#### Distribution.

Vietnam (Lam Dong), China (Yunnan).

#### Remarks.

*Unihamus elegans* (Hanitsch, 1927) was first described from Da lat, Lam Dong Province, Vietnam, and was subsequently recorded from Baoshan, Yunnan Province, China (Bey-Bienko 1957). Our specimens were collected in Jinping and Pingbian counties, Yunnan Province, which borders Vietnam; these specimens correspond to Hanitsch’s (1927) original description and to photographs of the holotype.

### Unihamus flavus

Taxon classificationAnimaliaBlattodeaBlattidae

Luo & Che sp. nov.

63334C33-8BBA-5503-99BB-F39D1740E9C0

https://zoobank.org/8E55BA7F-B38E-4F3A-81E8-893F4DB3B5BA

Figs 2A–D, 4, 7C, 7D, 8B, 8F, 8J, 9B

Unihamus sp. 1: Luo et al. 2025.

#### Type materials.

**Holotype**: China • ♂; Yunnan, Meizihu Reservoir, Pu’er City; 30.IV.2014; Xinran Li & Jianyue Qiu leg.; SWU-B-BL-082001. **Paratypes**: China • 1♀; Yunnan, Meizihu Reservoir, Pu’er City; 30.IV.2014; Xinran Li & Jianyue Qiu leg.; SWU-B-BL-082006 • 2♂♂; Yunnan, Dadugang Tea Garden and Secondary Plants, Xishuangbanna Prefecture; 1296–1299 m alt.; 28.IV.2014; Xinran Li leg.; SWU-B-BL-082002 to 082003 • 1♀; Yunnan, Meizihu Reservoir Highway, Pu’er City; 20–21.V.2016; Lu Qiu & Zhiwei Qiu leg.; SWU-B-BL-082004 • 1♀; Yunnan, Meizihu Reservoir, Pu’er City; 1384–1418 m alt.; 2014; Hongguang Liu & Xinran Li leg.; SWU-B-BL-082005 • 1♀; Guangxi, Yinshan Park, Jinxiu County; 16–17.VII.2015; Lu Qiu & Qikun Bai leg.; SWU-B-BL-082007.

#### Diagnosis.

Vertex slightly exposed. Pronotum subelliptical, widest posterior to midpoint. Posterior margin of metanotum mesally produced. Tegmina and wings well developed. Posterior branch of R not reaching end of tegmina. Hind metatarsus longer than or nearly equal to remaining tarsomeres combined. First abdominal tergite with visible gland. Hind margin of supra-anal plate mesally incised. Hind margin of supra-anal plate incised and divided into two subobtusely triangular lobules. Hind margin of subgenital plate slightly concave. L1 weakly sclerotized. L2 with a long ventral spine. L3 not bifurcate. L4C lanceolate, with distal part sharp and curved, with strip-like process near basal right margin. Left margin of R1H extended into a small spine; middle part of distal margin with a small projection. Distal margin of ventral surface of R1G extended into a long spine and another short spine.

Although closely resembling *U. elegans*, this species can be distinguished by the following combination of characters: 1) hind margin of supra-anal plate without small, sparse spines (Fig. 4F); 2) ventral surface of supra-anal plate without projections (Fig. 4F); 3) L4C with strip-like process near basal right margin and distal part curved (Figs 4H, 7C). In addition, *U. flavus* can be distinguished from *U. concavus* and *U. longispinus* by the following characteristics: 1) hind margin of supra-anal plate slightly concave (vs deeply concave) (Fig. 4F); 2) L2 with ventral spine shorter than *U. concavus* and *U. longispinus* (Figs 4H, 4I, 7C); 3) R1H broader than *U. concavus* and *U. longispinus* (Figs 4H, 7D).

#### Description.

***Colouration***. Body blackish brown to brown. Ocelli white, antennae yellowish brown. Tegmina reddish brown. Legs brown to reddish brown. Abdominal ventral surface unevenly coloured: margin black, middle reddish brown to yellowish brown.

**Male**. Body medium-sized. Total length: 26.1–29.5 mm; body length: 20.8–21.4 mm; pronotum length × width: 4.7–5.0 × 6.4–7.7 mm; tegmina length × width: 23.5–25.3 × 6.4–7.7 mm. ***Head and thorax***. Vertex slightly exposed; interocular space wider than interocellar space and shorter than interantennal space (Fig. 4B). Pronotum subelliptical, with anterior margin slightly concave and hind margin convex, surface smooth, widest posterior to midpoint (Fig. 4A). Posterior margin of metanotum mesally produced; posterior-lateral angles with symmetrical projections (Fig. 8B). Tegmina and wings well developed, surpassing tip of abdomen (Figs 2A, 2B, 4D, 4E). Tegmina ScP slightly thickened; posterior branch of R not reaching end of tegmina (Fig. 4D). Front femur of type A_2_ (Fig. 4C). Hind metatarsus longer than or nearly equal to remaining tarsomeres combined; metatarsi with two rows of spinules ventrally; each side of pulvillus with one spinule. Pulvilli present; claws symmetrical and unspecialized; arolium medium-sized. ***Abdomen***. First abdominal tergite gland with downward-pointing setae (Fig. 8B). Lateral margin of supra-anal plate contracted inward near distal part; median of hind margin incised and divided into two subobtusely triangular lobules (Fig. 4F). Paraprocts (pp.) elongate. Cerci strong and symmetrical. Subgenital plate overall nearly square; posterior lateral angles extended outward; hind margin of interstylar slightly concave (Fig. 4G). ***Genitalia*** (Figs 4H, 4I, 7C, 7D, 8F). L1 small and weakly sclerotized (Figs 4H, 7C). L2 irregular, with a ventral spine (Figs 4H, 7C). L3 unciform and well sclerotized, not bifurcate, with apex sharp (Fig. 8F). L4C lanceolate and broad, with strip-like process near basal right margin; distal part sharp and curved (Figs 4H, 7C). R1H broad and irregular; left margin extending into a small spine and middle part of distal margin with a small projection (Figs 4H, 7D). R1G well sclerotized; distal margin of ventral surface extending into a long spine and another short spine (Figs 4I, 7D).

**Female** (Figs 2C, 2D, 8J, 9B). Body slightly broader than male. Total length: 17.9–27.2 mm; body length: 17.9–27.2 mm; pronotum length × width: 4.7–6.2 × 6.3–8.0 mm; tegmina length × width: 15.3–21.2 × 5.3–8.4 mm. ***Head and thorax***. As in male. ***Abdomen***. Hind margin of tergum X with median invagination and a membrane line inside (Fig. 9B). ***Genitalia*** (Fig. 9B). First valve with small punctures. First valvifer slender and slightly curved. Second valve strip-like. Third valve large and wrapped around second valve. Posterior lobes of valvifer II less sclerotized; outer margin connected to laterosternite IX. Laterosternite IX broad and membranous. Paratergites long, strip-like, and slender. Anterior arch membranous, with dense microtrichia. Spermathecal plate hyaline, connected to basivalvula by membrane. Spermathecal opening at middle of basivalvula. Spermatheca branched, with duct long and curved and end capsule reniform (Fig. 8J). Basivalvulae nearly fan-shaped and symmetrical, membranous; surface with microtrichia. Laterosternal shelf symmetrical and membranous; surface with sparse punctures.

#### Etymology.

The species epithet is derived from the Latin word *flavus*, “yellow”, referring to the yellowish-brown ventral surface of the species’ abdomen.

#### Distribution.

China (Guangxi, Yunnan).

#### Remarks.

In their phylogenetic analysis, Luo et al. (2025) placed this species as the sister lineage of *U. elegans*. DNA barcoding supported the separation of these two species, revealing a genetic divergence of 5.92–6.26%.

### Unihamus concavus

Taxon classificationAnimaliaBlattodeaBlattidae

Luo & Che sp. nov.

74A47139-EBC1-5D28-8C68-EC5AEB0CD09E

https://zoobank.org/73D88A78-1A00-49A9-92FF-EE1F80217922

Figs 2E–H, 5, 7E, 7F, 8C, 8G, 8K, 9C

Unihamus sp. 2: Luo et al. 2025.

#### Type materials.

**Holotype**: China • ♂; Hainan, Mt Wuzhishan; 18–20.V.2014; Shunhua Gui & Xinran Li leg.; SWU-B-BL-083101. **Paratypes**: China • 1♀; Hainan, Jianfengling National Forest Park; 24.IV.2020; Yong Li & Jing Zhu leg.; SWU-B-BL-083102 • 1♂; Hainan, Jianfengling National Forest Park; 11–22.IV.2011; Wenxuan Bi leg.; 07002189 (Shanghai Entomological Museum, Chinese Academy of Sciences).

#### Diagnosis.

Vertex slightly exposed. Pronotum subelliptical, widest posterior to midpoint. Posterior margin of metanotum mesally produced. Tegmina and wings well developed. Posterior branch of R reaching end of tegmina. Hind metatarsus longer than remaining tarsomeres combined. First abdominal tergite with visible gland. Hind margin of supra-anal plate deeply concave inwards, W-shaped. Posterior lateral angles of subgenital plate extended outward; hind margin slightly concave. L1 weakly sclerotized. L2 with two long ventral spines. L3 not bifurcate. L4C elongate and distal part sharp and curved, and with spine-like process near basal right margin. Basal part of R1H with an elongate spine; distal margin with two spines. Distal margin of ventral surface of R1G extended into a strong spine and another slender spine.

This species can be distinguished from its congeners by the following characters: 1) supra-anal plate of male W-shaped (Fig. 5F); 2) hind margin of subgenital plate convex and middle part concave (Fig. 5G); 3) end of sclerite L2 with two long spines (Figs 5H, 5I, 7E); 4) R1H somewhat broad and basal part with an elongate spine (Figs 5H, 7F). In addition, *U. concavus* can be distinguished from *U. elegans* and *U. longispinus* by the curved distal part of L4C (Figs 5H, 7E).

#### Description.

***Colouration***. Body reddish brown. Ocelli white. Abdominal ventral surface reddish brown to blackish brown.

**Male**. Total length: 29.8–31.5 mm; body length: 23.71–24.0 mm; pronotum length × width: 5.64–6.0 × 7.89–10.1 mm; tegmina length × width: 24.6–25.5 × 8.1–9.2 mm. ***Head and thorax***. Vertex slightly exposed; interocular space wider than interocellar space and shorter than interantennal space (Fig. 5B). Antennae longer than body. Pronotum subelliptical, with anterior margin straight and hind margin slightly convex, surface smooth, median slightly elevated, widest posterior to midpoint (Fig. 5A). Posterior margin of metanotum mesally produced; posterior-lateral angles with symmetrical projections (Fig. 8C). Tegmina and wings well developed, surpassing tip of abdomen (Figs 2E, 2F, 5D, 5E). Tegmina ScP slightly thickened; posterior branch of R approaching end of tegmina (Fig. 5D). Front femur of type A_2_ (Fig. 5C). Hind metatarsus longer than remaining tarsomeres combined; metatarsi with two rows of spinules ventrally and with one spinule on each side of pulvillus. Pulvilli present; claws symmetrical and unspecialized; arolium medium-sized. ***Abdomen***. First abdominal tergite gland with downward-pointing setae (Fig. 8C). Lateral margin of supra-anal plate contracted inward; middle of hind margin deeply concave inwards, W-shaped; margin of hind margin with a row of long setae (Fig. 5F). Paraprocts (pp.) elongate, distally protruding downward. Cerci strong and long. Subgenital plate overall nearly square; posterior lateral angles extended outward; hind margin of interstylar slightly concave; styli slender (Fig. 5G). ***Genitalia*** (Figs 5H, 5I, 7E, 7F, 8G). L1 small and weakly sclerotized (Figs 5H, 7E). L2 irregular, with one long dorsal spine and another long ventral spine (Figs 5H, 7E). L3 unciform and well sclerotized, not bifurcate, with apex sharp (Fig. 8G). L4C elongate, with spine-like process near basal right margin and distal part sharp and curved (Figs 5H, 7E). R1H rather broad and irregular; basal part with an elongate spine; distal margin of broad sclerite with a large spine and a small spine (Figs 5H, 7F). R1G well sclerotized; distal margin of ventral surface extending a strong spine and another slender spine (Figs 5I, 7F).

**Female** (Figs 2G, 2H, 8K, 9C). Body slightly broader than male. Total length: 26.38 mm; body length: 23.71 mm; pronotum length × width: 6.01 × 8.44 mm; tegmina length × width: 19.4 × 6.85 mm. ***Head and thorax***. As in male. ***Abdomen***. Hind margin of tergum X with median invagination and with a membrane line inside (Fig. 9C). ***Genitalia*** (Fig. 9C). First valve with small, sparse punctures. First valvifer slightly sclerotized. Second valve strip-like. Third valve large and wrapped around second valve. Posterior lobes of valvifer II less sclerotized; outer margin connected to laterosternite IX. Laterosternite IX broad and irregular. Paratergites long, strip-like, and slender. Anterior arch broad, with sparse microtrichia. Spermathecal plate integrated into basivalvula. Spermathecal opening located at middle of basivalvula. Spermatheca branched, with duct rather short and end capsule rod-shaped (Fig. 8K). Basivalvulae nearly fan-shaped and symmetrical; surface with sparse microtrichia. Laterosternal shelf symmetrical, with sparse punctures.

#### Etymology.

The Latin word *concavus*, meaning “concave”, refers to the deeply concave hind margin of the supra-anal plate.

#### Distribution.

China (Hainan).

#### Remarks.

Pairwise COI distances between this species and the other three congeners range from 6.54% to 8.69%, supporting the establishment of this species based on DNA barcoding.

### Unihamus longispinus

Taxon classificationAnimaliaBlattodeaBlattidae

Luo & Che sp. nov.

128C5E34-F413-5B40-966F-CDAFDDDF5545

https://zoobank.org/206021C1-6BE3-466C-8D55-BEFF849EE846

Figs 2I–L, 6, 7G, 7H, 8D, 8H, 8L, 9D

#### Type materials.

**Holotype**: China • ♂; Hainan, Ganshiling, Baoting County; 9–10.IV.2015; Lu Qiu & Qikun Bai leg.; SWU-B-BL-083601. **Paratype**: China • 1♀; Hainan, Diaoluoshan National Forest Park; 2.V.2013; Shunhua Gui & Yan Shi leg.; SWU-B-BL-083602.

#### Diagnosis.

Vertex exposed. Pronotum subelliptical, widest posterior to midpoint. Posterior margin of metanotum mesally produced. Tegmina and wings well developed. Posterior branch of R reaching end of tegmina. First abdominal tergite with visible gland. Hind margin of supra-anal plate deeply concave inwards, with a row of long setae. Hind margin of subgenital plate slightly concave. L1 weakly sclerotized. L2 with one long, strong ventral spine. L3 not bifurcate. L4C lanceolate, with spine-like process near basal right margin. Basal part of R1H with a sharp spine. Distal margin of ventral surface of R1G extends into a strong spine and another slender spine.

This species can be distinguished from *U. concavus* by the shape of the supra-anal and subgenital plates (Fig. 6F, G). In *U. concavus*, the supra-anal plate is W-shaped and the subgenital plate is convex towards the back with the middle concave. It differs from *U. elegans* and *U. flavus* in having L2 longer than L3 and in having R1H spine-like (Figs 6H, 6I, 7G, 7H).

#### Description.

***Colouration***. Body brown to blackish brown. Ocelli white. Tegmina of male reddish brown and of female dark blackish brown to black.

**Male**. Total length: 24.0 mm; body length: 19.3 mm; pronotum length × width: 4.98 × 6.99 mm; tegmina length × width: 21.3 × 7.26 mm. ***Head and thorax***. Vertex exposed; interocular space wider than interocellar space, shorter than interantennal space (Fig. 6B). Antennae longer than body. Pronotum subelliptical, anterior margin straight and hind margin slightly convex, smooth, and widest posterior to midpoint (Fig. 6A). Posterior margin of metanotum mesally produced; posterior-lateral angles with symmetrical projections (Fig. 8D). Tegmina and wings well developed, surpassing tip of abdomen (Figs 2I, 2J, 6D, 6E). Tegmina ScP slightly thickened; posterior branch of R approaching end of tegmina (Fig. 6D). Front femur type of A_2_ (Fig. 6C). Hind legs unknown. Pulvilli present; claws symmetrical and unspecialized; arolium medium-sized. ***Abdomen***. First abdominal tergite gland with downward-pointing setae (Fig. 8D). Lateral margin of supra-anal plate contracted inward, with median of hind margin deeply concave inwards, and with a row of long setae (Fig. 6F). Paraprocts (pp.) elongate. Cerci strong and long. Subgenital plate overall nearly square; hind margin of interstylar slightly concave; styli slender (Fig. 6G). ***Genitalia*** (Figs 6H, 6I, 7G, 7H, 8H). L1 weakly sclerotized (Figs 6H, 7G). L2 irregular; end with a long, strong spine (Figs 6H, 7G). L3 unciform, well sclerotized, and not bifurcate (Fig. 8H). L4C lanceolate, with spine-like process near basal right margin; distal part sharp (Figs 6H, 7G). R1H irregular; basal part with a sharp spine (Figs 6H, 7H). R1G well sclerotized; distal margin of ventral surface extended into a strong spine and another slender spine (Figs 6I, 7H).

**Female** (Figs 2K, 2L, 8L, 9D). Body slightly broader than male. Total length: 23.55 mm; body length: 21.29 mm; pronotum length × width: 5.9 × 8.3 mm; tegmina length × width: 18.8 × 8.0 mm. ***Head and thorax***. As in male. ***Abdomen***. Hind margin of tergum X with median invagination, and with a membrane line inside (Fig. 9D). ***Genitalia*** (Fig. 9D). First valve with small punctures. First valvifer rather broad. Second valve sclerotized. Third valve large and wrap second valve. Posterior lobes of valvifer II connected to laterosternite IX. Laterosternite IX broad and irregular. Paratergites strip-like and slender. Anterior arch broad; surface with sparse microtrichia. Spermathecal plate fan-shaped and integrated into basivalvula. Spermathecal opening at middle of basivalvula. Spermatheca branched, with duct short and end capsule rod-shaped (Fig. 8L). Basivalvulae symmetrical; basal part of lateral margin contracted inward; surface with sparse microtrichia. Laterosternal shelf symmetrical and with sparse punctures.

#### Etymology.

The species epithet is derived from the Latin word *longispinus*, meaning “long spined”, in reference to L2 ventral spine in males, which is longer than in congeneric species.

#### Distribution.

China (Hainan).

#### Remarks.

Pairwise COI distances between this species and the other three congeners range from 5.73% to 7.65%. The genetic distance between *U. longispinus* and *U. flavus* is 5.73%, which is less than the genetic distance between most species. However, they strongly differ in their morphology.

##### Molecular analysis based on COI

**Figure 10.**
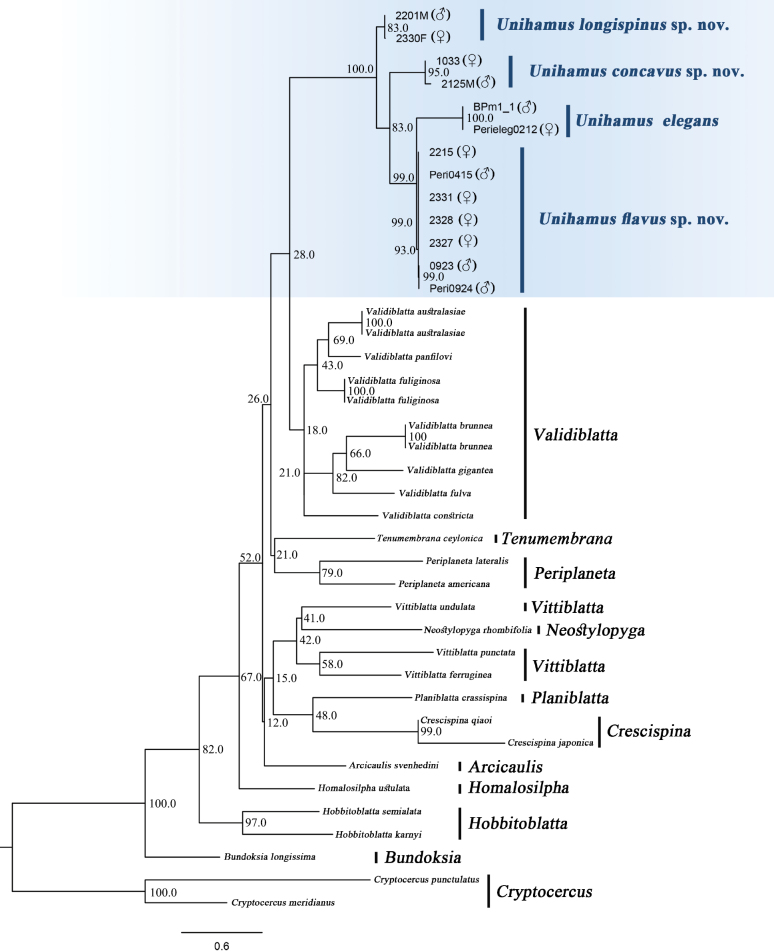
Maximum-likelihood tree derived from COI sequences with 10,000 ultrafast bootstrap replicates.

Two COI sequences were obtained from *U. elegans*, *U. longispinus* Luo & Che, sp. nov., *U. concavus* Luo & Che, sp. nov., and seven COI sequences were generated for *U. flavus* sp. nov. All new sequences were deposited in GenBank with accession numbers PZ151070 to PZ151078. In our maximum-likelihood (ML) analysis, the *Unihamus* species each formed a well-supported monophyletic group (Fig. 10), including female and male specimens. Intraspecific COI genetic divergence (K2P) was 0.00% in *U. elegans*, 0.00–0.30% in *U. flavus* sp. nov., 0.46% in *U. longispinus* sp. nov., and 1.85% in *U. concavus* sp. nov. Interspecific K2P distances between the four species ranged from 5.73% (*U. flavus* sp. nov. and *U. longispinus* sp. nov.) to 8.69% (*U. elegans* and *U. concavus* sp. nov.) (Suppl. material 2).

## Discussion

*Unihamus*, *Validiblatta*, *Arciaulis*, *Tenumembrana*, and *Crescispina* were formerly included in *Periplaneta* s.l. because of their similar external morphology. Phylogenomic evidence presented by Luo et al. (2025), however, demonstrated that these genera are distantly related. Therefore, the traditional classification of Blattinae based primarily on conserved external morphology did not accurately reflect the phylogenetic relationships. Luo et al. (2025) also reconstructed the evolution of male genitalia in Blattinae, revealing that sclerite L3 of *Unihamus* is distinctly different from that of other genera of Blattinae. However, their study did not describe the detailed genital characters of this genus. In the present study, we associate the sexes of *Unihamus* by using COI barcoding and provide comprehensive descriptions of external morphology and genitalia for both males and females of each species studied. These data will enable rapid and reliable identification of the genus and supply baseline information for future functional analyses of genital sclerites.

Genital sclerites in cockroaches are notoriously complex (McKittrick 1964; Klass 1997), yet their functional significance has been elucidated for only a few elements. Sclerite L3, in particular, is thought to anchor the female genitalia during copulation. In *Blattella germanica* (Linnaeus, 1767), the hook hla with sclerite L3 fixed the female basivalvulae during mating (Bell et al. 2007; Khalifa 1950), and in *Blatta orientalis* Linnaeus, 1758, the same structure was found to be the first male appendage to contact to the ovipositor (Bao and Robinson 1990). In contrast, L3 is absent in the giant burrowing cockroaches *Macropanesthia rhinoceros* Saussure, 1895 and *M. heppleorum* Walker, Rugg & Rose, 1994; its loss is interpreted as evidence for type-III mating behaviour, in which the male backs onto the female to initiate copulation (Roth 1971, 1977; Bell et al. 2007). Among blattine genera with described male genitalia, all possess a bifurcate L3, comprising hlat and hlap, except for *Unihamus*, whose L3 lacks hlap. However, the function of the hlap in mating is unknown. Reconstruction of the ancestral state indicates that hlap was secondarily lost in *Unihamus* (Luo et al. 2025). Whether this loss is stochastic or linked to a shift in mating behaviour requires further investigation.

## Supplementary Material

XML Treatment for Unihamus

XML Treatment for Unihamus elegans

XML Treatment for Unihamus flavus

XML Treatment for Unihamus concavus

XML Treatment for Unihamus longispinus

## Additional information

### Use of AI

No use of AI was reported.

### Author ORCIDs

Xin-Xing Luo https://orcid.org/0009-0000-6838-1696

Meng-Yu Qiu https://orcid.org/0009-0006-1555-7794

Ting-Ting Cai https://orcid.org/0009-0007-7796-2514

Zong-Qing Wang https://orcid.org/0000-0001-9413-1105

Yan-Li Che https://orcid.org/0000-0003-3214-9494

### Data availability

All of the data that support the findings of this study are available in the main text or Supplementary Information.

## Supplementary materials

Supplementary material 1 Samples used in ML analyses with abbreviation, GenBank ID, identifier, and collecting information https://doi.org/10.3897/zookeys.1277.163465.suppl1 Data type xls https://binary.pensoft.net/file/1597591This dataset is made available under the Open Database License (http://opendatacommons.org/licenses/odbl/1.0/). The Open Database License (ODbL) is a license agreement intended to allow users to freely share, modify, and use this Dataset while maintaining this same freedom for others, provided that the original source and author(s) are credited. Xin-Xing Luo, Meng-Yu Qiu, Ting-Ting Cai, Zong-Qing Wang, Yan-Li Che

Supplementary material 2 Genetic divergence of distances calculated by K2P model method using cytochrome c oxidase https://doi.org/10.3897/zookeys.1277.163465.suppl2 Data type xlsx https://binary.pensoft.net/file/1597592This dataset is made available under the Open Database License (http://opendatacommons.org/licenses/odbl/1.0/). The Open Database License (ODbL) is a license agreement intended to allow users to freely share, modify, and use this Dataset while maintaining this same freedom for others, provided that the original source and author(s) are credited. Xin-Xing Luo, Meng-Yu Qiu, Ting-Ting Cai, Zong-Qing Wang, Yan-Li Che
